# The development of a CBT-informed approach to supporting type 2 diabetes self-management

**DOI:** 10.3389/fcdhc.2025.1421678

**Published:** 2025-02-05

**Authors:** Elné Visagie, Elmari Deacon, Rümando Kok

**Affiliations:** ^1^ Compres Research Focus Area, Psychology, Faculty of Health Sciences, North-West University, Potchefstroom, South Africa; ^2^ Department of Psychology, University of Pretoria, Faculty of Humanities, Pretoria, South Africa; ^3^ Optentia Research Unit, Psychology, North-West University, Potchefstroom, South Africa; ^4^ Centre for Health and Human Performance, Faculty of health Sciences, North-West University, Potchefstroom, South Africa

**Keywords:** type 2 diabetes, self-management, cognitive behaviour therapy, CBT, CBT informed guidelines, adults

## Abstract

**Background:**

The self-management of adults with type 2 diabetes proves to be a continuous challenge. Within the South African context, socio-economic disparities, unequal access to healthcare and varying healthcare beliefs augment these challenges. CBT is a time-sensitive and structured intervention that has been effectively implemented for chronic diseases. CBT has been employed to improve psychological outcomes in adults with type 2 diabetes, but there is limited research on how this therapeutic intervention can enhance self-management outcomes of type 2 diabetes. Adaptable CBT-based interventions are needed to promote a holistic approach to type 2 diabetes self-management and empower a broader range of healthcare professionals to provide targeted interventions. Flexible interventions can promote patient engagement and be integrated into various healthcare settings where patients already access services.

**Method:**

The study employed document analysis to develop CBT-informed guidelines. Data was analysed by means of content analysis. Two research studies formed the dataset, and categories and subcategories were identified. The categories were integrated and used to develop the CBT-informed guidelines.

**Results:**

These guidelines were based on core CBT components and divided into the introduction, working, and consolidation phases. The guidelines specified the roles of healthcare practitioners who would implement them and provided skills and techniques for healthcare professionals and patients within each phase.

**Conclusion:**

The CBT-based interventions aimed to provide a tool for healthcare practitioners and patients in terms of flexibility, accessibility, and personalisation.

## Introduction

Type 2 diabetes (T2DM) has become increasingly prevalent, specifically in developing countries where a lack of resources poses a significant challenge to healthcare systems and global health initiatives (1). T2DM accounts for 90% of diagnosed cases and places individuals at higher risk for developing comorbid conditions and diabetes-related complications (2). Receiving a diagnosis of T2DM requires individuals to take on new roles and responsibilities to manage their condition. The demands of the condition often contribute to feelings of distress, and individuals find that their thoughts revolve around the limitations of their diagnosis and possible existing and future complications (3). This poses a burden for individuals as they must engage in multifaceted decision-making, incorporate lifestyle changes into established routines and remain vigilant for any diabetes-related complications (4, 5).

Adding to the complexity of navigating this chronic condition is that self-management forms the cornerstone of diabetes management (6). Self-management encompasses a set of behaviours that needs to be steered by the individual to achieve glycaemic control and enhance their quality of life (1). This proactive and pragmatic approach involves a repertoire of tailored activities aimed at medical management (adherence behaviours), role management, and emotional management (3). In a broader context, self-management is an essential practice to enhance patient adherence, promote health-related behaviours, and foster greater autonomy (7). This allows for improved symptom management that augments self-efficacy and overall engagement with self-management (1).

Self-management proves challenging, with approximately 50% of individuals reporting poor glycaemic control (8). This statistic increases in developing countries due to cultural variabilities, different socio-economic status, and resource constraints (8). Abbas et al. underscored the need for interventions that specifically focus on improving self-management (9). Interventions that focus on imparting knowledge and skills regarding self-management can instill self-confidence in individuals pertaining to their diabetes management and enhance efficacious engagement in self-management behaviours (6, 9).

As an evidence-based intervention, cognitive behavioural therapy (CBT) has shown promise as it goes beyond behavioural skills impartation but also considers and addresses the cognitive conceptualisation of the condition and equips individuals with cognitive, emotional, and behavioural knowledge and skills (9). CBT is a structured and feasible therapeutic intervention that underpins the relationship between thoughts, emotions, and behaviours (10). The process of behavioural change and the mechanisms promoting these adjustments are viewed through a cognitive lens, highlighting that thoughts influence emotional responses and health-related behaviours (11). Experiencing a thought such as *controlling my diabetes is impossible* evokes adverse feelings such as anger and helplessness, ultimately leading to undesirable health-related behaviours (1). Employing CBT can orientate individuals to their thoughts about self-management and heighten awareness of the interplay between glycaemic control, negative thought patterns, behavioural reactions, and emotional responses (1). CBT theories offer a theoretical foundation for targeted interventions, emphasising altering maladaptive thought processes as the basis of the intervention, followed by establishing effective behavioural change (11). This approach holds the potential to furnish healthcare providers with the knowledge and a structured framework to target impeding management-related thoughts, emotions, and behaviours while facilitating effective problem-solving and self-efficacy for patients. This fosters greater patient engagement and prompts individuals to take a more proactive role in their self-management (4). Furthermore, by equipping a range of healthcare practitioners who work with individuals managing T2DM, especially in developing countries, healthcare systems can expand access to mental health support and chronic disease management (12). This approach can improve accessibility, foster improved health outcomes and enhance patients’ resilience, particularly in settings where resources are limited (12).

There have been numerous studies about CBT and its efficacy in diabetes management and self-management practices (9). Vlachou et al. conducted a review and found that most studies included in the review reported improved glycaemic control following CBT interventions (4). Similar findings were reported by Li et al. and Jenkinson et al. (13, 14). Koochaksaraee et al. corroborated and elucidated these findings, explaining that CBT taught participants to alter irrational thoughts, beliefs, attitudes, and behaviours, which promoted increased engagement and self-efficacy (1).

Li et al. reported that interventions aimed at modifying health behaviours for desired management outcomes have progressively gained prominence as a central area of investigation (13). There has been a shift in research focusing on the conventional application of CBT. Gobin et al. found that current research has started to examine the alternative formats of CBT interventions with the aim of enhancing their accessibility (15). Thus, this study set out to develop CBT-informed guidelines that followed CBT’s theoretical basis and structure but allowed healthcare practitioners the flexibility in implementation to promote patient-centred intervention, accessibility, and the consideration of context-specific needs.

## Materials and methods

### Study design

A qualitative approach employing document analysis was conducted in the research study. Document analysis is a systematic procedure for reviewing, evaluating, and interpreting data to elicit meaning, gain understanding, and develop empirical knowledge (16, 17). Document analysis allowed the researchers to actively discover, collect, and make meaning of existing and generated knowledge (17). This entailed the examination of two key documents that formed part of a doctoral thesis, which aimed to develop a greater understanding of self-management. It enabled the researcher to explore self-management through the CBT paradigm to ultimately develop and evaluate CBT-informed guidelines to facilitate self-management practices of adults with T2DM. The two documents were critically examined, serving as the dataset for the development of the CBT-informed guidelines. The execution of this research study adhered to overarching ethical principles. Ethical approval was obtained from the (institution omitted for blind review), reference number NWU-00301-21-A1.

### Data collection

Two studies’ empirical findings were used as data sources. Collecting information from different research sources strengthened the applicability and quality of the CBT guidelines.

#### Document 1

This article employed a rapid review to identify and integrate current scientific research regarding CBT-based interventions that focused specifically on the self-management of individuals with T2DM (18). The review identified nine articles that fit the inclusion criteria and delineated the structure and content of CBT interventions. Two main themes with respective sub-themes were identified, namely (1) the characteristics of the CBT-based interventions, which included the format, duration, and outcomes, and (2) identifying the techniques and components used in the CBT-based interventions, which included establishing rapport, psychoeducation, working with cognitions, identifying, and managing emotions, facilitating behavioural change, homework, social support and creating an action plan. This knowledge was significant in determining evidence-based practices and tailoring interventions to suit the unique needs of individuals with T2DM. The process of delineating the structure, content, and specific skills and techniques was required to identify the foundational structure and approach of CBT interventions and use this information to structure and develop the CBT-informed guidelines.

#### Document 2

This article explored the self-management-related thoughts, emotions, and behaviours of adults who manage their T2DM effectively and those who struggle with management (19). An explorative qualitative design with seventeen semi-structured interviews was employed to collect data. Eight participants struggled with self-management (HbA1c>10%) and nine participants engaged in effective self-management (HbA1c<8%). The following themes were identified: (1) emotional experience, (2) prominent cognitions, (3) practising acceptance, and (4) the mechanisms of behavioural change. This document was considered essential as it may provide insights into the thoughts, emotions, and behaviours experienced by adults with T2DM that need to be targeted in the CBT guidelines to enhance self-management practices.

### Data analysis

The data was analysed by means of content analysis, which has also been referred to as a method of analysing documents (20). Content analysis embedded in a qualitative framework is the process where large amounts of data are categorised to summarise the key findings (21, 22). This analysis method underlined the aim of the current study as it enabled the researchers to distil the dataset into a more concise set of content-related categories and sub-categories. Inferences were made from the categories and were used to develop the CBT-informed guidelines. As the development of the guidelines covered the implementation process and which content to include, both the data’s manifest (literal meaning) and latent content (underlying meaning) were analysed. The data analysis encompassed an inductive approach, as the study aimed to formulate CBT-informed guidelines within the context of self-management for type 2 diabetes.

Initially, the researcher engaged in data immersion and endeavoured to comprehend the dataset. A co-coder independently reviewed the two documents. Open coding was employed, and consequent headings and categories were formulated. The process of immersion revealed cognitive, emotional, and behavioural codes in the dataset. Moreover, it became evident that CBT-based interventions followed a specific implementation method, providing a scaffold of knowledge, skills, and techniques. These categories were extensively reviewed and discussed through expert consultations and collaboration with the co-coder. This iterative process refined and amalgamated categories encompassing individuals’ thoughts, emotions, and behaviours in self-management, along with categories related to CBT skills and techniques. Additionally, the structure of the guidelines was identified. These findings were integrated in terms of format, the guidelines’ process, the content of thoughts, emotions, and behaviours and the skills required to address this and evolved into CBT guidelines for self-management among adults with type 2 diabetes.

Steps to ensure methodological rigour and trustworthiness in each document included bracketing and reflexivity. The researchers in all the studies were adequately trained to gather and analyse the data. Utilising these different sources of information cultivated triangulation, which enhanced trustworthiness through the validation of findings across multiple studies, thereby mitigating potential biases inherent in a single inquiry (16, 23).

## Results

**Figure 1:**
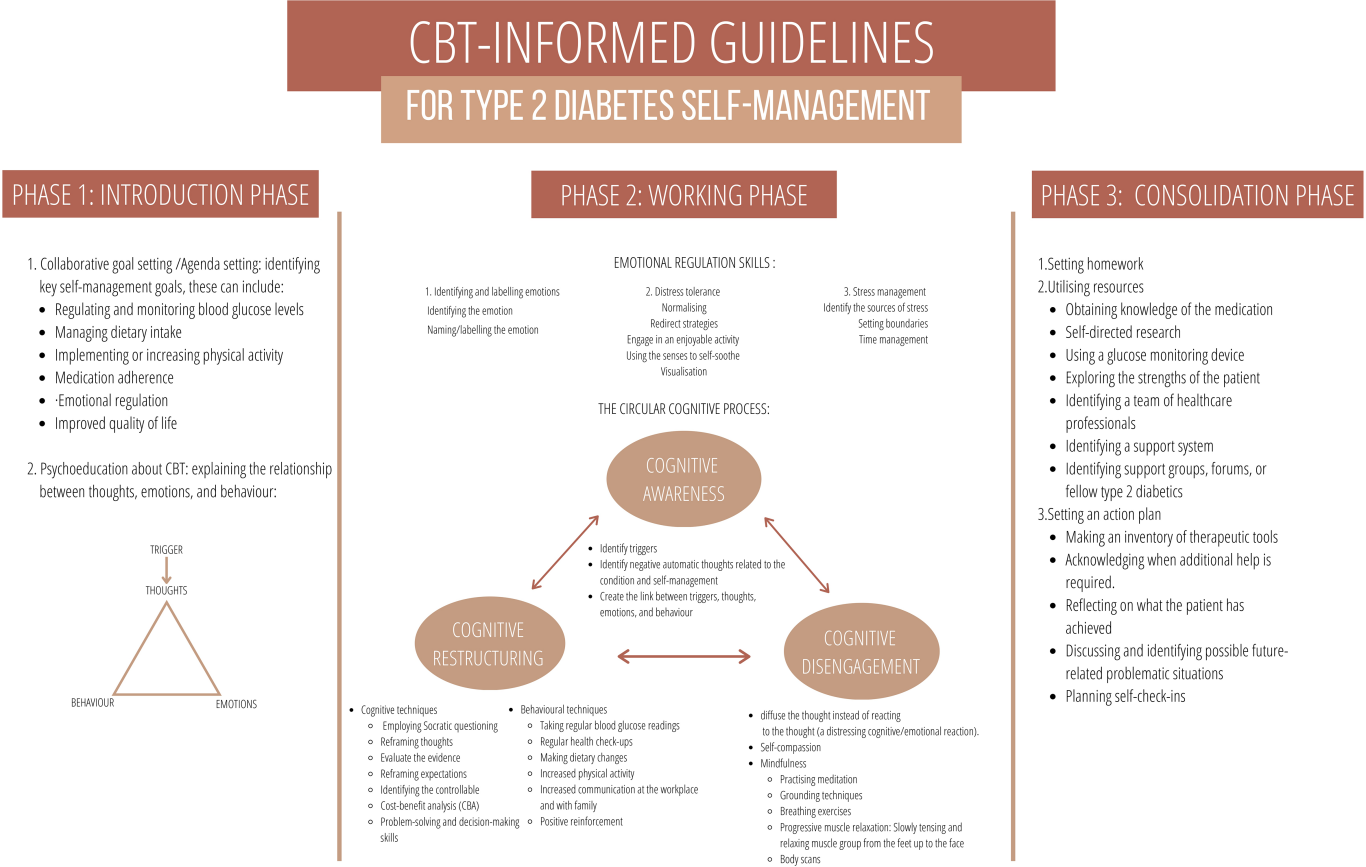
A visual representation of the CBT-Informed Guidelines.

The study employed content analysis to develop the CBT-informed guidelines. The categories identified three main phases, each with a subset of skills and techniques, as represented by **Table 1**. The three phases were the introduction phase, the working phase, and the consolidation phase. The categories and sub-categories were integrated to develop the CBT guidelines, represented by **Figure 1**. Preceding the phases, the guidelines provided a preface for practitioners, delineating the guidelines’ aims, the responsibilities of the healthcare practitioners, and a brief description of the three phases.

**Table 1:** Summary of the CBT-informed guidelines content categories and subcategories.

Phases of the CBT-informed guidelines
1. The introduction phase
1.1. Providing psychoeducation
1.2. Collaborative goal setting
2. The working phase
2.1. Emotional regulation skills
• Identifying and labelling emotions • Distress tolerance • Stress management
2.2. The circular cognitive process
• Cognitive awareness • Cognitive restructuring • Behavioural techniques • Cognitive disengagement
3. The consolidation phase
3.1. Setting homework
3.2. Utilising resources
3.3. Setting an action plan

### The introduction phase

The introduction phase aimed to accustom individuals to CBT and how it relates to their self-management practices. This phase was solidified in the data obtained from Documents 1 and 2. Document 1 reported that establishing rapport was the cornerstone of effective intervention and led to improved adherence and self-efficacy (11, 24–30). Document 2 (19) revealed that patients felt heard and understood when their physicians were attuned to their needs. Strengthening the individual’s commitment to adapting their self-management was providing psychoeducation. Psychoeducation regarding CBT, self-management-related practices, and diabetes-specific knowledge was essential. This was corroborated by Document 2, which found that obtaining information aided effective self-management. Finally, goal setting was highlighted by both Documents 1 (31) and 2 to enhance motivation and foster responsibility taking enhance motivation and foster responsibility-taking as participants felt included in the decision-making process.

### The working phase

The working phase addressed the emotions, thoughts, and behaviours by suggesting specific skills and techniques and providing diabetes-specific examples.

#### The emotional component: emotional regulation skills

Document 1 laid the groundwork and identified valuable emotional regulation skills to use within the guidelines. Emotional regulation was described as a process through which individuals had to identify, monitor, evaluate, and modify the emotion experienced. Techniques identified in Document 1 included creating awareness of emotions, practising being present, grounding techniques, meditation, various breathing exercises, body scans, and identifying pleasant situations (11, 24, 25, 27–30).

Document 2 supplemented this information and provided insights into the different emotions that adults experienced, making them more relatable to individuals with type 2 diabetes (19). This was characterised by denial and shock initially. Thereafter, it ranged between frustration, anger, guilt, and worry. The valuable insight that Document 2 provided was that people who effectively manage T2DM generated motivation and positive emotions by meeting self-management goals and experiencing self-management victories. Individuals who struggled tended to ruminate on the negative emotions, echoing hopelessness and helplessness, which hindered their ability to regulate their emotions. The guidelines, therefore, aimed to provide individuals with emotional regulation skills to navigate challenging emotions and positive reinforcement to facilitate the generation of positive emotions.

#### The cognitive component: the circular cognitive process

The circular cognitive process highlighted the process of becoming aware of one’s thoughts to realise their impact and adjust these perceptions and influences accordingly. Data obtained from Documents 1 and 2 significantly informed this phase of the guidelines. Data from Document 1 stressed working with cognitions and set out a process of cognitive awareness, cognitive restructuring, and cognitive disengagement (11, 24–30). Cognitive awareness facilitated individuals to become aware of their thoughts; cognitive restructuring aided the development of alternative thoughts, and cognitive disengagement aimed to change the relationship with the thoughts. These cognitive processes were prominent in the experiences of the participants in Document 2. They aided the guidelines in showing how the relationship and restructuring of one’s thoughts facilitated or impeded self-management (19). The three cognitive approaches can help HCPs identify which cognitive skill their patient requires and employ the relevant skill set.

#### The behavioural component: behavioural techniques

Document 2 was pivotal in providing insights into the process of behavioural change. The process described by participants progressed from *why I need* to self-manage to *what I need to do* and *how to do it*, highlighting the importance of patient engagement with their process of change (19). This resulted in improved knowledge, self-confidence, and improved skill acquisition to improve self-management. Document 1 suggested behavioural techniques such as behaviour modifications, behavioural rehearsal, and coping skills (11, 24–31). Document 2 provided diabetes-specific behavioural modification. Effective behavioural modifications and coping skills that were included in the guidelines were the following:

- Identifying and utilising resources such as identifying social support structures (strongly emphasised in Document 1) and obtaining information through healthcare practitioners, peers, and self-directed research (also highlighted under the consolidation phase) (11, 24, 26–31).
- Developing an awareness of the body’s responses. Through their awareness and understanding of their body’s reaction, participants were able to take corrective measures, experience a greater sense of control, and feel secure in their ability to determine when to make dietary adjustments or abstain from specific foods (19).
- The participants experimented with different foods to see how their bodies reacted (19).
- Identifying healthier alternatives enabled participants to enjoy a range of foods without feeling deprived (19).

### The consolidation phase

Document 1, which explored key components of CBT interventions (11, 24, 26–30), strongly embedded the consolidation phase. A fundamental CBT principle is setting homework, identifying resources, and creating an action plan for future challenges. It allows individuals to practise skills and techniques learned and develop the ability to monitor and acknowledge small victories and identify alternative ways to manage future difficulties.

The process of identifying resources was required throughout the working and consolidation phase. Resources are embedded in a session but also facilitate homework and action plans by equipping the individual with internal and external coping strategies. Document 2 highlighted the resources utilised by participants, which included internal and external resources (19). HCPs can use these resources to identify what thoughts individuals are struggling with and then identify which cognitive skills to employ to assist their patient’s self-management.

A valuable insight from Document 2 was that individuals who managed their T2DM effectively viewed themselves as a resource, which promoted self-efficacy and autonomy.

## Discussion

The CBT-informed guidelines aimed to provide a set of techniques to healthcare practitioners to facilitate the self-management practices of the patients that they encounter in their clinical settings. Incorporating the psychological aspect into the medical intervention for T2DM can address the current challenges faced by individuals (32). The guidelines were constructed based on fundamental CBT principles but were not intended as a therapeutic intervention; their format and method of delivery were adapted accordingly. Research suggests that using the community of health providers to increase knowledge and provide interventions for T2DM in under-resourced communities empowers both the practitioners and the individuals seeking treatment (18). Having practitioners be more equipped with diabetes related thoughts, behaviours, and emotions; and techniques to approach this allows for more affordable and culturally relevant mental health care in developmental settings and creates greater accessibility to individuals as limited funding, restrained resources and inadequate access to a team of healthcare professionals (12, 19). This can promote self-management practices and patients who might otherwise lack access to specialized mental health services (12, 18).

The introduction phase was designed to establish rapport and introduce CBT by means of psychoeducation. Koochaksaraee et al. reported that psychoeducation regarding type 2 diabetes, self-management, and CBT provide a basis of knowledge to the patient, facilitating patient engagement and promoting effective self-management (1). Orientating individuals to the relationship between thoughts, emotions, and behaviours enabled them to efficiently convey their thoughts, feelings, wants, and needs. This improved understanding of their approach to self-management, fostering greater motivation for change (9). Subsequent to establishing a framework of knowledge regarding CBT and self-management, collaborative goal-setting could take place. Collaborative goal setting considers the patient’s needs, preferences, and contexts, promoting enhanced self-management (33). It underscores the person-centred nature of CBT interventions, further promoting adherence (34).

The working phase aimed to equip patients with emotional, cognitive, and behavioural skills to improve self-management. The importance of emotional regulation and fostering motivation was central to the emotional skills provided in the guidelines. Incorporating these skills was highlighted by Powers et al., who reported that practising emotional regulation skills facilitated decision-making, problem-solving, and interpersonal effectiveness, allowing individuals to navigate and make improved decisions regarding their self-management (35). Javidan et al. reported that emotional regulation and positive reinforcement promoted greater engagement with self-management and enhanced self-efficacy, which facilitated continuous prioritising of health and thus maintained effective self-management (36).

Cognitive skills were central to the development of the guidelines, which emphasised cognitive awareness, cognitive restructuring, and cognitive disengagement. Li et al. and Velázquez-Jurado et al. supported that CBT techniques, such as introducing the cognitive triangle (enhancing cognitive awareness) and cognitive restructuring, enabled participants to recognise their thought patterns and cognitive distortions to improve distorted and negative thinking (3, 13). Similarly, findings reported by Abbas et al. corroborated that cognitive restructuring facilitated adaptive thinking patterns in individuals with T2DM (9).

Thoughts cannot always be altered or changed; therefore, cognitive disengagement was incorporated into the guidelines. Cognitive disengagement forms part of the third wave of CBT techniques, which incorporates skills to change individuals’ relationship with their thoughts instead of changing the content. Jenkinson et al. conducted a systematic review and reported that third-wave techniques, such as the cultivation of acceptance, mindfulness meditation and cognitive diffusion, reduced emotional distress regarding self-management (14). Accordingly, the guidelines incorporated techniques that facilitated cognitive disengagement.

Fundamentally, the guidelines proposed collaboration between healthcare practitioners and patients, as this proves to be a central component of promoting health-related behaviours and active decision-making (6). Powers et al. stated that the relational foundation and engagement of the patient are the cornerstone of behavioural change (35). Information sharing, problem-solving and self-monitoring underpin behaviour change, allowing patients to identify alternative options, direct their course of action and feel confident in their decision-making (35). In accordance, the introduction phase and the cognitive circular process served as the basis for behavioural change by providing skills and techniques that fostered patient engagement. Behavioural techniques incorporated into the guidelines that have been proven to promote behaviour change include goal setting, behavioural rehearsal, problem-solving, social support, self-monitoring, and action planning (37). Employing these behavioural activities, individuals experienced an enhanced sense of competence in managing their condition, ultimately reducing hindering behaviours (14).

The consolidation phase’s purpose was to promote sustained self-management by enhancing self-efficacy and autonomy. The guidelines implemented techniques such as identifying external supportive resources, setting homework tasks to practise skills, or formulating a comprehensive action plan for anticipated challenges. This aligns with the aims of the CBT guidelines, which aspire to equip individuals with relevant knowledge, emotional skills and cognitive techniques that can enhance self-efficacy (9). Findings by Abbas et al. and Dong et al. supported that implementing homework assignments and setting up a plan for future setbacks allowed individuals to practise the skills and techniques they acquired (7, 9). Homework activities strengthened adherence to adequate nutrition, regular physical activity, and blood glucose monitoring and promoted self-efficacy (13). Self-efficacy played an essential role in formulating and maintaining restructured thoughts and the maintenance of self-management-related behaviours, as participants developed the ability to monitor and acknowledge small victories and identify alternative ways to manage future difficulties (38).

The skills and techniques incorporated in the three phases align with core CBT components and structure. The structure of the guidelines is intended for HCPs to navigate the different phases to choose the relevant information and skills that the patient requires and tailor the implementation of the guidelines. Providing this flexibility, the guidelines aim to accommodate the diverse and dynamic nature of patients’ needs, preferences, and circumstances and ultimately facilitate enhanced emotional regulation, glycaemic control, and self-management practices (4).

## Conclusions: strengths and limitations

The guidelines offer a promising and accessible framework for multiple healthcare practitioners to use with their patients. Similarly, they offer individuals with type 2 diabetes the opportunity to develop and practise skills that can facilitate self-management outcomes. Incorporating core CBT elements without rigid time limitations and structure allows for a more patient-centred approach to self-management while promoting mental health. The guidelines aim to foster collaboration between healthcare practitioners and their patients to identify skill deficiencies and individualised needs, fostering a more targeted and personalised intervention. The guidelines allow mental health to be incorporated into medical health and promote a holistic approach to diabetes management.

There are some limitations to be considered. Further research is required to explore and understand how these guidelines can be used in the South African context. Randomised controlled trials are needed to explore the efficacy of the guidelines and determine their applicability within the South African context. Future research needs to investigate patients’ and healthcare professionals’ responsiveness to the guidelines. Especially exploring in which contexts the guidelines prove to be effective and which patients find the non-traditional format beneficial. Furthermore, the flexibility of the guidelines can lend itself to a strength and a limitation. There is a need for standardised CBT interventions with regard to T2DM self-management, and further investigations are needed to determine effective intervention length, number of sessions, duration of sessions and the required training for individuals implementing CBT-based interventions.

Finally, the guidelines provide an introductory framework for exploring non-traditional formats of CBT. Future research is suggested to explore the potential of diverse implementation by experimenting with the efficacy of expanding the guidelines into a group intervention, digital programme, and therapeutic intervention specifically for T2DM.

## Data Availability

The raw data supporting the conclusions of this article will be made available by the authors, without undue reservation.
